# The Choice and Use of Medical Literature

**Published:** 1905-02

**Authors:** Hugh T. Patrick

**Affiliations:** Chicago, Ill.


### SELECTIONS AND ABSTRACTS.

### The Choice and Use of Medical Literature.*

By Hugh T. Patrick, M.D., Chicago, Ill.

*Ladies and Gentlemen:* From the beginning, the Mississippi Valley Medical Association has been blessed above other scientific societies with a general friendliness—indeed, a fraternal affection—among its members. Second only to the American Medical Association in size and in geographical extent, second to none in earnest and fruitful work, our Association has retained a sort of family warm-heartedness unique in large bodies. For this we should be thankful. For this your President is peculiarly grateful, for well he knows that to this kindly spirit and to no merit of his own he owes his present honorable position. During his brief incumbency it shall be his great pleasure as well as duty to foster the old friendships and cement the new.

And now, since inexorable by-laws require you to undergo something called an address, I am going to inflict a few desultory remarks about the matter and the manner of our reading.

The choice and use of medical literature will depend first of all upon what the physician tries to be; not upon what he would like to be, not upon what his ideal may be, but upon what he thoughtfully, consistently, persistently strives to be.

Our day-dreams of attainment are much the same, but the daily walk of doctors varies greatly. Most of us have met a doctor who may be called the family factotum. He hob-nobs with fussy mothers and puttering fathers. He is greatly interested in grandma's cough, knows just how to wash the baby, has his special poultice, and can take off warts. Dropping in to ask about Aunt Em's backache, he stays an hour visiting with the folks. He is a gentle and kindly soul, but his mind is occupied with the trivialities of medicine and domestic chit-chat.

---

*President's Address, delivered before the thirtieth annual meeting of the Mississippi Valley Medical Association, at Cincinnati, October 11, 1904.

Then there is our old friend, who in winter airs his surgical deeds and medical acumen about the drug store stove and in summer holds a like symposium on the shady side of the street. He knows a good cigar, is a pleasant gentleman and harmless in all things, save only the practice of his profession.

We have heard of the affable society doctor who dresses well, talks well, knows the best families, attends receptions and makes social calls. We know and respect the God-fearing church doctor who teaches the Bible class, sings in the choir, gets together the pastor's salary, and finances the church debt. The lodge doctor, the sporting doctor, and the doctor deep in politics are familiar figures. We know them all. They are of us, members of our honorable guild. With loyalty and affection we take them by the hand. They are good friends and good citizens; but really fine physicians? Not one of them. And not one of them has any real use for good medical literature.

But there is another man: The man we would like to be; the capable man who knows his work and does it; the man up with the van, who can talk face to face with the great in our noble profession. He has enthusiastically striven or doggedly persisted until he is the wise, skillful physician who practices with no uncertain hand, but knows when he is right and knows where he is ignorant. To be what he is and do what he does means familiarity with what others have done and are doing. To this there is no exception. He reads. He selects his literature well and uses it wisely.

The selection of our books is a task as delicate, a duty as significant as is the choice of remedies for our patients. Every book is a prescription for our mental self. And yet who can deny that the clever book agent puts thousands of volumes upon our shelves? In this day and generation it is the doctor's obligation not only to be able to recognize the good in medical literature, but to know the great and the safe among medical writers. A conscientious physician calls in consultation no unknown man, but, strange to relate, he will blindly follow an author of whom he knows nothing except that he has produced a book.

Another somewhat frequent professional indiscretion, to call it by no harsher name, is the good old student plan of sticking to a textbook. Progress means expansion. The more a man progresses the less promnent is the role of the text-book. It is necessary for babes, but not meat for strong men. No text-book doctor is an A No. 1 man. The latter needs the treatise, the "system," the cyclopedia, and particularly the monograph.

Of all medical books the monograph is the best—and the worst. As a rule it is the product of special interest and wise employment of opportunity. In it we often find the rich harvest of many years of loving labor by a great mind. In all probability the author has not only consulted the writings of others, but has acquired a sure judgment in weighing their merits. Unfortunately, sometimes he is an enthusiast without balance, a faddist, a man with a theory, a prejudiced observer, a dealer in sophistry. Then, if in addition he be a positive and clever writer, he may do untold harm. I have now in mind an interesting, attractive book which in the last twelve years has led astray thousands of physicians, and I am sure that the next quarter of a century will not see rooted out the fallacies planted by its brilliant author. Why is this? Because thousands of well-meaning doctors bought the book without knowledge, read it without care, absorbed its tenets without proving and promulgated its dicta without prudence.

If he is to really profit by the precious time spent in reading, the physician must be able to read with discrimination. In medicine there is no such thing as an authority. The critical sense must be keen and ever alert. The reader must learn to be a judge, for the plea of every paragraph is to be adjudicated. And if he feels himself at fault, assistance is on every side. I think there is no case in which those who know are more happy to lend a hand than in the selection and interpretation of medical writings.

And next, what of the journals? This is a big question and a hard one; one I approach with considerable feeling, but no confidence. Nevertheless, I shall not attempt to dodge it. And since to advise is more human than to confess, to find fault more spontaneous than to praise, to say "don't" easier than to say "do," I venture first to advance a few of the "don'ts" in my mind. But be it understood that these are suggestions purely tentative, the expression of only one of a vast company, and that no remark is meant for criticism of editor, censure of publisher or condemnation of publication *per se*. All is addressed to the reader. I am not talking of foods, but of diet.

Don't admit to your presence a journal that is not perfectly straight and clean. As the first requisites of the good physician are conscientiousness and strictest integrity, so no journal is safe if it be not honest on every page. Humiliating as is the admission, we must confess that there are mercenary medical men writing subsidized articles for the benefit of tradesmen. We may not be able to stop the practice, but for the love of decency let us drop the articles into the waste-basket as soon as received and bar the journals from our table.

Just to illustrate how vigilant we must be, I may state that one medical screed has appeared in different journals as a straight advertisement, an editorial, an "original," a therapeutic hint, a news item and a clinical note. Again, of seven so-called original papers in one journal, four were obviously for ulterior purposes.

A thinly veiled deception is that of putting amidst scientific matter blatant advertising quoted from *Der Deutsche Medizinische Schurkenstreich* or *La Nation Medicale Trompeuse.* It is simply knavery with German or French sugar-coating.

Another sort of debasing journal is the one that pretends, perhaps honestly, to help the physician to success by means other than pure professional excellence. A type of this kind of thing I recently found in an address to the graduating class of a medical school. I have no doubt the orator thought he was advising tact and inculcating practical methods for the management of patrons, the confusion of competitors and the increase of income. As a matter of fact he was teaching those young men deception, subterfuge and meretricious connivance, to take the place of scientific knowledge and manly worth. Concerning the editor who spreads such pestilence I have nothing to say. The doctor who admits it to his library and his mind, not only injures himself but is recreant to his trust.

Don't indulge in yellow journals—for such there are, of deeper or fainter dye. The more harmless kinds merely present a sort of pseudo-scientific vaudeville of striking oddities, rare curiosities, marvelous happenings and other side-show monstrosities of the medical world. All of this serves only to excite a passing interest and makes no reader one whit better as scholar or practitioner. The worse kinds, under the cloak of medicine, pander to our appetite for the startling, the scandalous, even the salacious, and appeal to passion and prejudice. They lean strongly to sexual perversion and suggestive gossip. Behind the mask of independent thought they delight in strictures on those high in the profession, and even indulge in dirty inuendo. They start hot discussions on ethics and and foment controversies over personal rights, privileges and immunities. And this, God save the mark, is circulated as medical literature because it is read by medical men.

Don't take a journal which is run as an advertising medium, and don't look at it if it is sent to you. Such publications are of two sorts. One kind is issued by some commercial gentleman to assist in selling his wares. In the guise of a scientific periodical it is to all intents and purposes a sort of medical almanac about as wholesome and edifying as the pamphlet for Bonesetter's bitters or the bulletin of Mother Udder's uterine uplifter. The other kind is conducted by a medical man with the dollar mark stamped on his aspirations. The good of the reader is no concern of his. He is after the money of advertisers, all of them. Original communications, editorials, excerpts, correspondence, everything is arranged purely as a bait to induce the gullible to swallow the ads.

Don't read a journal that accepts abortive papers by underdone doctors. There are journals which systematically encourage that sort of thing. Unquestionably, to report cases and write down his opinions is good for any physician. But how about the reader? When the cases are incompletely studied, the writer ignorant or narrow, his conclusions lacking foundation and his judgment immature, the contribution is not only valueless, it is injurious. Smooth is the descent that leads to Avernus and easy the downward road of this damning third-rate literature. It is light and easy reading, but begets self-satisfaction, blunts the critical sense, lulls ambition, dulls observation, stunts mental growth, and before he knows it the reader is found on a low plane of thought and practice—no higher than the twaddle he reads.

Don't waste time on journals abounding in short cuts. They are an abomination unto the mind, a snare for the unwary, and their name is legion. A hint or two will indicate the sort I mean.

Purporting to be practical and immediately helpful, some journals make a specialty of what may be called recipes for disease. And they are very alluring. Instead of learning all about pneumonia, its nature, course, variations and complications; what methods of treatment have been tried and abandoned and what has been the experience of those seeing hundreds of cases, it is so much easier to take some fellow's or some journal's statement that a peculiar poultice or ambitious alkaloid cures the disease. Stamped deep on my feelings is a paper on dyspepsia in a journal of great vogue. With no statement as to what dyspepsia may be, with no word as to diagnosis, with no allusion to pathology, no mention of gastroptosis, dilatation, hyperacidity, or motor power, and no hint of test-meal or examination of stomach contents, the author proceeded to advise the administration of seven different drugs. In spite of the multifarious remedies, such advice simplifies practice to a degree. It is no task simply to remember to give this tonic for appetite, that capsule for digestion, this granule for pain, those drops for nausea, one pill for constipation, the other for diarrhea, and the powder for flatulence. It is a short cut, but it leads to disaster.

In this same category belong the medical magazines that make a leader of questions and answers—a department modeled on the *Ladies' Fireside Guide* where anxious inquirers go off at half-cock and the answers pop back as prompt and empty as echoes. A very little reflection will show not only the utter futility of this kind of reading, but how it prevents development by curtailing wholesome mental effort.

Then there is the petty journal corresponding to the family factotum above mentioned. Its short cut is simply the avoidance of the great and profound in medicine. Ignoring such fundamental things as anatomy, physiology and pathology, oblivious alike to basic principles and the best of accumulated experience, it propagates a sort of family confab on the various superficialities of practice, How to bring out the eruption of measles; what is good for hiccough; the best liniment for sprains; these are cheerfully aired in numerous columns. What do you say to the parturient woman when she grows impatient? What is your favorite catarrh snuff? In the treatment of "threatened" appendicitis should aconite be given the first day and veratrum the second, or *vice versa?* Upon questions such as these the editor and his writing readers expend great energy, priceless time and endless ink. With great zest the contributors, as Charles Lamb says, encourage one another in mediocrity. I carefully went through 115 pages of such a penny-wise pound-foolish publication and found just six pages of good stuff. And yet that journal has an enormous circulation—to the great renown of the publishers and the great discredit of the medical profession.

Don't pay much attention to columns of formulæ, notes on treatment, therapeutic hints, brief paragraphs on recent discoveries, and items on new drugs. Pass over abstracts in which the process of condensation has squeezed the life out of the matter and skip society reports so meagre as to amount to mere personal mention. Most of such matter is garbled at the best, has no educational value, and even when a bit of .it has virtue, is pretty sure to just slip through the otic tunnel—in one ear and out the other.

From the foregoing negatives a few positives may readily be inferred. As we are to buy only the best books, so let us take only high-class medical periodicals. And then let us read them well. If there are many poor journals there is much poor reading done—reading that is casual, unsystematic, careless, superficial, cursory, profitless. We have three sources of information and inspiration—personal experience, personal contact with colleagues, and reading. The first may be limited, the second unsatisfactory, but the last offers to all a most bountiful supply of both inspiration and knowledge. Then let us read up and not down.

There is a certain comfortable ease in reading what we already understand. We may gratify our natural craving for approval by reveling in nice little papers which repeat what we have been saying for years. To tickle our vanity by reading papers so poor that they show the author to be more ignorant than ourselves is a pleasing process. Sometimes we feel quite luxuriously virtuous when reading a medical journal purely for entertainment and mental relaxation. None of these things should be. One and all they create a slovenly habit of mind. They are destructive of good method and in the end incapacitate us for good work.

Here I must notice an objection or complaint that we have all heard and most of us have made: "I have no time to read." It isn't true. The apportionment of time is not a matter of necessity, but of choice. What do I consider of the greater importance? What do I like and dislike? What do I *choose* to do? These are the determining questions. And in the modern physician's life there is precious little paramount to study. It *pays* to read. Besides the pleasure of knowledge and the power that knowledge gives, besides the gratifying sense of achievement and the satisfaction of progress toward a goal, it pays in dollars. In conversation with the busiest, the greatest and the most successful of our colleagues I have often been astonished at the amount of reading they do and how they rely upon it. Very, very often good reading makes the difference between $5.00 and $25.00 for an examination, between $50.00 and $500.00 for an operation.

And now, if I may be allowed four little hints as to the manner of reading, I shall have finished.

One excellent way to use medical literature is systematically to get up one subject well; to investigate it thoroughly; to trace its history and follow its development; to scrutinize diverse observations, review conflicting opinions and weigh different conclusions. Having once mastered the thing, it will be surprisingly easy to follow it through the succeeding years, for the annual increment to any given subject in medicine is astonishingly small. After one topic is exhausted another may be attacked, and so onward.

This method easily falls in with a second good one, namely, to read up fully on cases in hand. Note that I say *fully*. Hastily to look up an ointment or eczema for consult a text-book or two on the diagnosis of iritis may be one of the exigencies of practice; it is not reading. Likewise hunting up a remedy for an obscure case may possibly be a necessary makeshift, but generally it does the patient little good and the doctor less. We should read for a perfect understanding of the case, which means a complete comprehension of the subject. No case can properly be considered alone. It is always in relation to variant cases under diverse circumstances. For no patient can there be a paragraph explanation and a recipe treatment.

Now these two plans of reading naturally lead us to a third—reading to write. Of course, this plan is good only when the writer compels himself to produce really good stuff. This proviso fulfilled, I know of nothing more wholesome than the writing of papers and the reading of them where others may pass judgment. If the first two plans have been well carried out it is reasonably certain that this one will not miscarry. The man who has excellently well worked up any subject or any case of any abnormal condition, is not only well prepared for the next one of kind, but he is in a position to tell others something they don't know.

Pursuance of these three plans of reading will almost invariably produce a most desirable habit in practice, viz: the keeping of case records The virtues of this practice are many. Two of them are that it stimulates reading and enhances its value. Accurate comparison of our experience with that of others not only serves to impress the facts, but ripens knowledge into wisdom.

The fourth and last suggestion for medical reading is really but a summary of the other three. It is that we acquire the mental attitude, or aptitude, or habit, of *reading for reproduction.* To be a student is not enough. We must be effective students—student soldiers, if you please, preparing for action. There is a vast difference between the acquisition of knowledge as a mere accomplishment and as a means of accomplishment. It is well for us doctors to regard our store of knowledge not as simply an interesting museum of Nature's wonders marvelous to contemplate, but rather as an armamentarium, an orderly array of goodly weapons ready for instant use.—*Cincinnati Lancet-Clinic.*

---

Mr. Steinwine: "Hello, Jakey, did you hear about Solomon Isaacs? He vas bad sick, and they took his apendix away from him."

Jacob: "Mein Gott! Vy didn't he put it in his vife's name?"

